# Fluid upstream shear stress of rabbit aortic stenosis inhibits neointimal hyperplasia by promoting endothelization after balloon injury

**DOI:** 10.1186/s12872-017-0690-3

**Published:** 2017-10-30

**Authors:** Jinxue Liu, Yucheng Peng, Junxing Lai, Weidong Gao, Anjian Song, Gaoxing Zhang

**Affiliations:** Department of Cardiology, Jiangmen Central Hospital, No.23, Haibang Street, Jiangmen, Guangdong 529030 China

**Keywords:** Hemodynamic, Neointimal hyperplasia, Shear stress

## Abstract

**Background:**

Atherosclerosis is associated with disturbed blood flow characterized by low and oscillatory shear stress (SS), however, few study directly links SS to neointimal hyperplasia in animal model. This study was focused on the effects of changed SS upon the neointimal hyperplasia which responded to balloon injury in a novel rabbit model with partially-constricted abdominal aorta.

**Methods:**

We established a rabbit model subjected to partial abdominal aortic constriction with a cylinder-shaped cannula as a model of disturbed flow, which was similar to the hemodynamic features of stenosis caused by atherosclerosis plaque. Further, balloon injury was performed to investigate the relationship between SS and neointimal hyperplasia. Four weeks later, the abdominal aorta was assessed with digital subtraction angiography (DSA) and intravascular ultrasound (IVUS). The vascular sections were embedded in paraffin blocks for morphometric analysis to evaluate neointimal hyperplasia, and anti-CD31 immunohistochemical staining was for endothelialization ratio.

**Results:**

In upstream the stenosis, the changed SS leads to neointimal hyperplasia compared with normal SS (11,729 ± 1205 *vs* 8418 ± 737, *P =* 0.023). However, the upstream SS of the stenosis can promote vascular re-endothelialization after balloon injury compared with normal SS, verified by endothelialization ratio (0.36 ± 0.03 vs 0.32 ± 0.03, *P =* 0.017), thereby attenuate neointimal hyperplasia (64,851 ± 3995 *vs* 68,335 ± 3867, *P =* 0.018).

**Conclusion:**

The upstream SS of stenosis, not downstream SS, inhibits the neointimal hyperplasia after balloon injury by promoting vascular re-endothelializtion.

## Background

Atherosclerosis and related complications are the leading cause of mortality in most countries. Though a lot of systemic factors, including such as dysglycemia, hypercholesterolemia, hypertension and smoking, are identified as risk factors, disturbed flow was more likely to play a pivotal role in atherosclerosis development with a nonrandom but preferentially pattern, which was characterized by low and oscillatory wall shear stress (SS) in branched or curved arteries [1, 2]. Increasing evidences have indicated that SS was the chief culprit in the development of vascular injury. With the applications of intravascular ultrasound (IVUS) and computational fluid dynamics, researchers demonstrated that SS could be used to predict in-stent restenosis-induced neointimal formation and assess post-balloon angioplasty vascular remodeling progress [3]. Research has shown that high SS is associated with in-stent restenosis and target lesion revascularization, even in successful balloon angioplasty cases [4]. However, the relationship between SS and neointimal hyperplasia induced by balloon angioplasty needs to be further confirmed.

Vascular endothelial cells (ECs) are the essential parts of the inner lining inblood vessel wall and always directly exposed to disturbed blood flow. Meanwhile, ECs could be influenced by various chemical and mechanical stimuli, which finally regulated homeostatic functions [5]. EC dysfunction is a vital pathology factor in vascular diseases, including atherosclerosis and thrombosis. In recent years, a variety of models in vitro have been established to regulate shear flow to ECs, which are aimed to mimic important features of ECs flow environments in vivo. These models helped researchers to explore detailed mechanisms of their responses to SS, including the effects on EC morphology, cytoskeletal organization, proliferation, migration, permeability and junctional proteins, EC signaling and gene expression [1, 4, 5].

Multiple studies have explored effect of SS on vascular structure in vitro [4, 5]. However, the role of SS in the vascular remolding and neointimal formation has not been completely clarified, especially disturbed flow in vivo. In addition, an appropriate model should be established to explore this issue. Thus, it is important for better clinical understanding to establish an animal model in which disturbed flow can be acutely induced. On the other hand, previous study indicated rabbits were ideal animals for vascular model [2]. Accordingly, we enrolled the rabbits subject to transverse abdominal aortic constriction with a cylinder-shaped cannula, which mimics important hemodynamic features of stenosis caused by atherosclerosis, to explore the generated SS on the effect of the vascular remolding after balloon injury in vivo. We hypothesized that this constrictive vascular model, similar to the atherosclerotic plaque, will cause varying degrees of intimal hyperplasia in the upstream and downstream of the constricted vessel, with or without balloon injury.

## Methods

### Animals

All experimental protocols complied with the guidelines for the Chinese Animal Care and Use Committee standards. All in vivo procedures were also performed in accordance with protocols approved by the Animal Ethics Committee of Sun Yat-sen University. 40 New Zealand white rabbits were purchased from cavenstech company (Changzhou, China, permission code for experimental animals: SCXK2016–0010), weighing 2.2–2.6 kg, male, were randomly divided into 5 groups (*n* = 8 per group), were housed individually in steel mesh cages and fed normal rabbit chow and water for at least 1 week prior to operative procedures. The rabbit was anesthetized with 3% pentobarbital (1 ml/kg) and then the right femoral artery was cannulated with a four introducer sheath. All catheters were subsequently introduced through this sheath and advanced to the abdominal aorta through a 0.014-in. guidewire. Heparin sodium (200 IU/kg) was intra-arterially injected. After that, a midsagittal incision was made and the middle vessel of the renal artery to the common iliac artery about 1 cm in length was separated, waiting for the next step which is decided by the grouping. Baseline angiogram and IVUS of the abdominal aorta were obtained for each rabbit. All operations were under the sterile condition.

### Experimental protocol

**Fig. 1:**
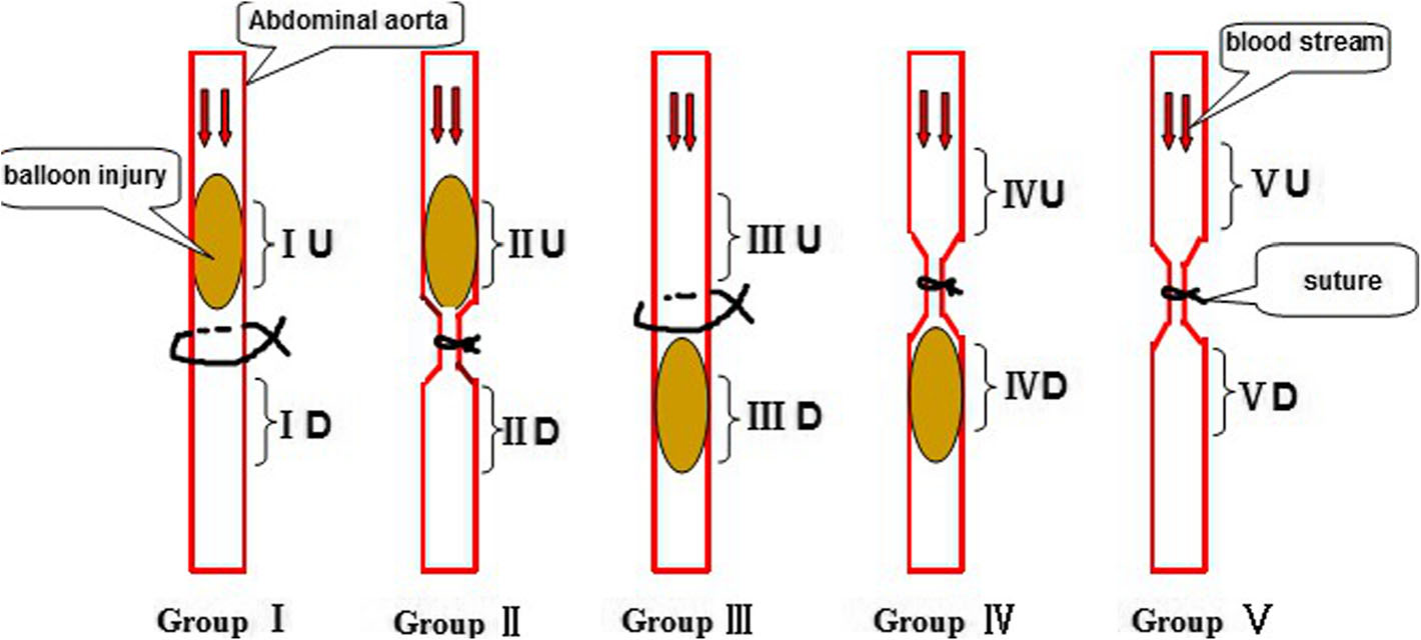
Schematic illustration of the experimental animal group. The rabbit abdominal aorta was balloon injured and/or partially constricted with a cylinder-shaped cannula by the surgical suture according to the grouping. Four weeks later, the upstream and downstream vessels were harvested and subjected to hematoxylin-eosin and immunohistochemistry staining. Group I (BU group) = the group of balloon injury the aorta just upstream the separated artery and put a surgical suture without ligation as control; Group II (BU + C group) = the group of balloon injury the aorta just upstream the separated artery and constrict the separated aorta; Group III (BD group) = the group of balloon injury the aorta just downstream the separated artery and put a surgical suture without ligation as control; Group IV (BD + C group) = the group of balloon injury the aorta just downstream the separated artery and constrict the separated aorta; C group = only constrict the aorta group. U = upstream, D = downstream

**Fig. 2:**
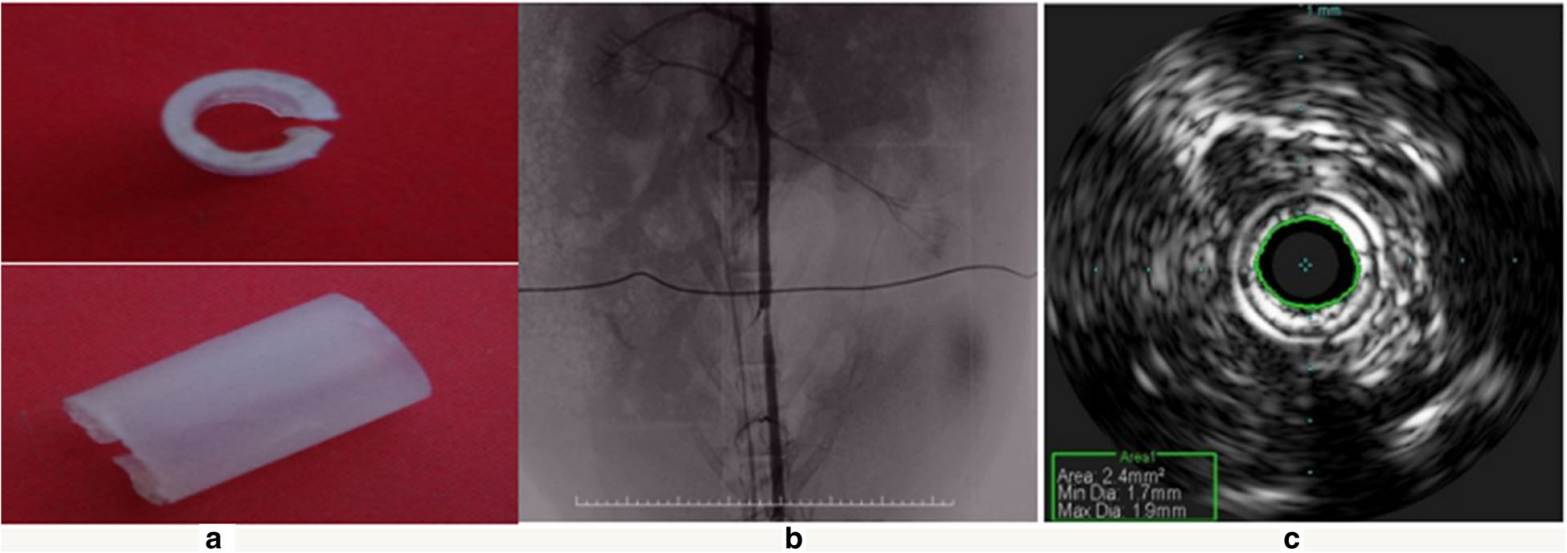
The cylinder-shaped cannula (**a**) using for the separated artery constriction and representative images of the DSA (**b**) and IVUS (**c**) at the site of the rabbit constricted abdominal aorta. The diameters of the constricted arteries are about 2 mm (**c**)

Operative procedures of every group are shown in Fig. 1. Group I: only balloon injury the aorta just upstream the separated artery and put a surgical suture without ligation as control. Group II: not only balloon injury the aorta just upstream the separated artery, but also partially constricted the isolated artery with a cylinder-shaped cannula (diameter about 2 mm, length 5 mm) (Fig. 2a), ligated by a surgical suture. Group III: only balloon injury the aorta just downstream the separated artery and put a surgical suture without ligation as control. Group IV: balloon injury the aorta just downstream the separated artery and partially constricted the isolated artery with the cylinder-shaped cannula, which ligated by a surgical suture. Group V: only partially constricted the separated artery with the cylinder-shaped cannula ligated by a surgical suture. At the level of the balloon dilatation injury, the expansion ratio of the aorta was between 1.2 and 1.3 according to the basic IVUS diameter data for twice in the 10-s interval manner at the same location, the digital subtraction angiography (DSA) and IVUS images were recorded (Fig. 2b, c). After all the operations, the incision was closed by suture under aseptic conditions and antibiotics were injected intravenously to prevent infection. The animals were allowed to recover and fed a normal diet after the intervention.

Four weeks later, the rabbit was checked with DSA and IVUS again and then euthanized with an overdose of pentobarbital, the aorta was dissected from the renal artery to iliac bifurcation was perfusion-fixed with 10% neutral formaldehyde overnight. The fixed aorta was embedded in paraffin blocks for morphometric and immunohistochemical studies.

### Morphometry

Morphometric analysis was performed with the Image-Pro Plus 6.0 image analyze system by an independent observer blinded to the study groups. Cross-sections were obtained from two different sites, the very upstream and downstream sections of the constricted artery or sham groups. The tissue samples were cut into serial 5 μm cross-sections for general histological staining with hematoxylin and eosin (HE) and Verhoeff-Van Gieson. Image-Pro Plus 6.0 image analyze system was performed to determine lumen area (LA, pixel) and internal elastic lamina area (IELA, pixel), and neointima area (NA, pixel) was calculated using the following equation: NA = IELA-LA. The mean area in the corresponding segment (*n* = 3 sections per segment) was used for statistical analysis.

### Immunohistochemistry

Immunohistochemistry for paraffin embedded cross sections was described previously [6]. In brief, after deparaffinization and hydration, sections were treated with 3% H_2_O_2_ in PBS for 10 min to inhibit intrinsic peroxidase activity and 5% blocking solution for 20 min to prevent non-specific antibody binding. Antigen retrieval was performed for cross sections with citrate buffer (pH 6.0). They were then incubated overnight at 4°Cwith CD31 monoclonal antibody (1:50; Abnova). After rinsing in PBS, slides were then incubated for 20 min at room temperature with goat anti-rat IgG, followed by 20 min for SABC complex. Slides were rinsed again with PBS and were stained with DAB, counterstained in hematoxylin, dehydrated, and mounted. Control immunostaining was carried out by the same procedure in which the primary antibody was replaced by nonimmunized serum.

### Statistical Analysis

The results of HE staining and immunohistochemical staining were examined by a professional pathological doctor using the Image-Pro Plus 6.0 image analyze system [5]. Five views on every slides were chosen randomly and the area density of positive expression (positive area/total area) was quantified. Results are described as mean ± SD. Statistical tests were performed with SPSS (version 13.0; SPSS Inc., Chicago, Ill), using Student t test for 2 groups or one-way ANOVA followed by multiple comparisons with Bonferroni test. Probability values of *P* < 0.05 was considered as significant.

## Results

### Establishment of Rabbit model

Four rabbits (two in group 2, one in each of the group 3 and group 4) died of excessive anesthesia, thrombosis, hemorrhagic shock, balloon injury during the experiment and the remaining rabbits completed the entire study. The average weight of rabbits 2.36 ± 0.25 kg at baseline but increase to 3.14 ± 0.16 kg four weeks later. There was no significant difference in body weight between the five groups (dates not shown), suggesting model was successfully established. Hemodynamic assessments were performed to confirm changing of vascular SS after partial abdominal aortic constriction. These routine measurements are assessed to confirm the model of changed SS.

### The upstream SS of stenosis attenuates the lumen loss after balloon injury

**Fig. 3:**
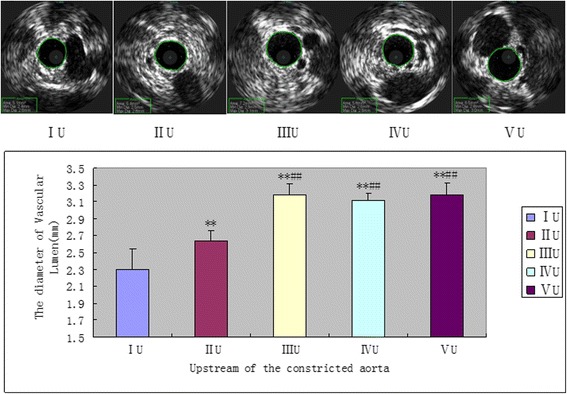
The upstream vascular diameter of the constricted aorta was measured by IVUS 4 weeks later. **P < 0.05 significantly different from the groupIU; ##*p* < 0.05 significantly different from the groupIIU.U = upstream

**Fig. 4:**
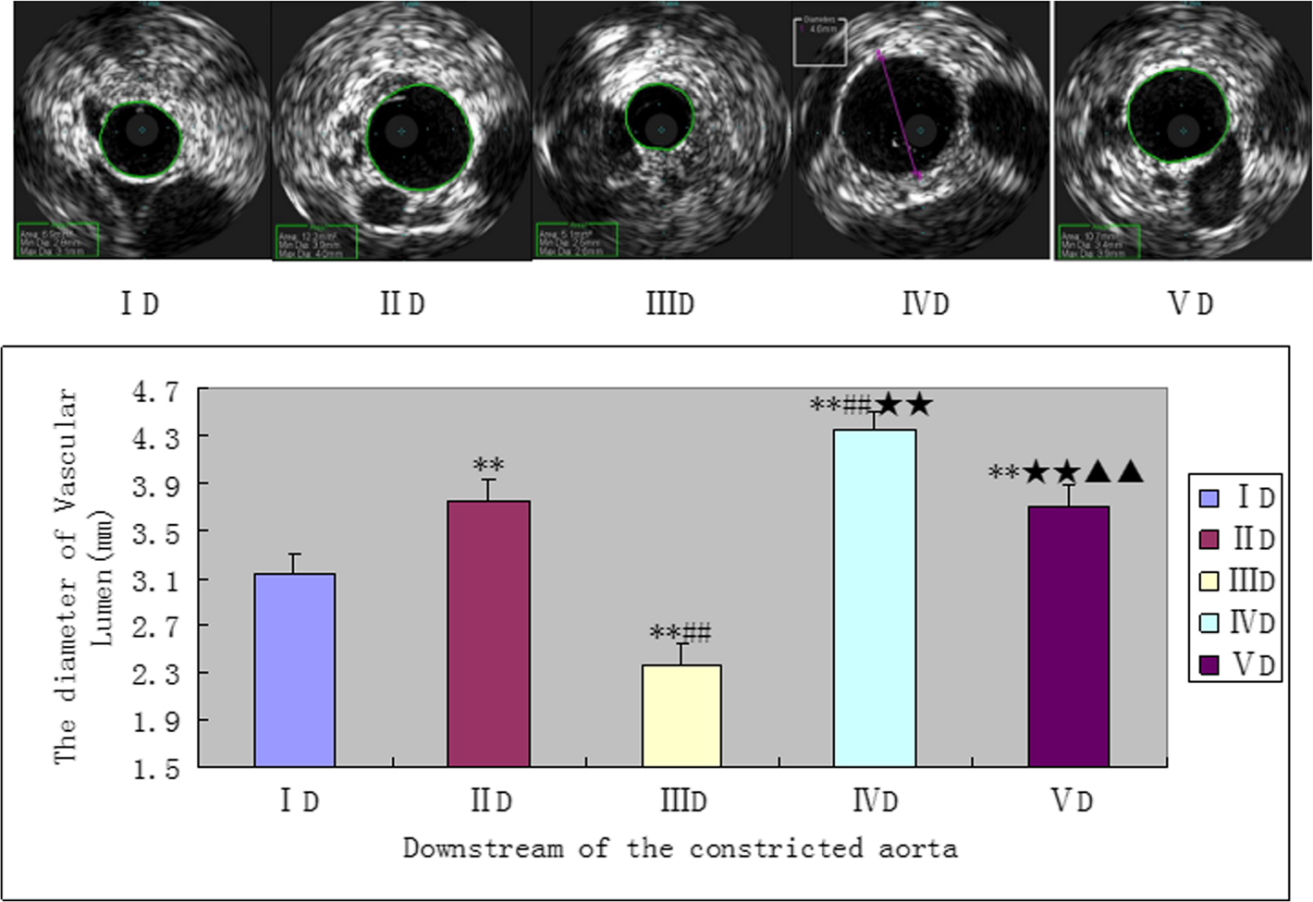
The downstream vascular diameter of the constricted aorta was measured by IVUS 4 weeks later. **P < 0.05 significantly different from the groupID; ##*P* < 0.05 significantly different from the groupIID;★★P < 0.05 significantly different from the groupIIID;▲▲*P* < 0.05 significantly different from the groupIVD.D = downstream

Four weeks later, the upstream and downstream vascular diameters of the separated arteries were measured by IVUS in order to evaluate the vascular remodeling which was secondary to the balloon injury and/or constriction. In the upstream of the separated artery, compared with other three no balloon injury and/or constriction groups, balloon injury significantly decreased the lumen diameter in the single balloon injury group (Group IU,2.3 ± 0.24 mm,^**^ *P* < 0.05) and balloon injury besides constricted group (Group IIU,2.6 ± 0.12 mm, Fig. 3, ^##^ *P* < 0.05). Furthermore, the artery constriction did not result in the further reduction of the inner diameter which was secondary to the balloon injury but attenuate the upstream vessel lumen loss (Fig. 3, Group IU 2.6 ± 0.12 mm *VS.* Group IIU 2.3 ± 0.24 mm, ^**^ *p* < 0.05).Meanwhile, compared with other groups, the single artery constriction didn’t cause a significant change in the vessel diameter compared with the normal vessel. In the downstream of the separated artery, the single balloon injury can significant decrease the lumen diameter in group IIID (2.4 ± 0.18 mm, Fig. 4,^★★^ *p* < 0.05), suggesting that constriction leads to an expansion of the normal vascular lumen in group IID (3.8 ± 0.18 mm) and groupVD (3.7 ± 0.19 mm, Fig. 4, ^##^ *P* < 0.05). Moreover, balloon injury makes the expansion tendency become more apparent in group IVD thus causing the vessel lumen has a excessive expansion (4.3 ± 0.15 mm, Fig. 4, ^▲▲^ *p* < 0.05).

### The upstream SS of stenosis induces neointimal hyperplasia in the normal vessel but alleviates the hyperplasia after balloon injury

**Fig. 5:**
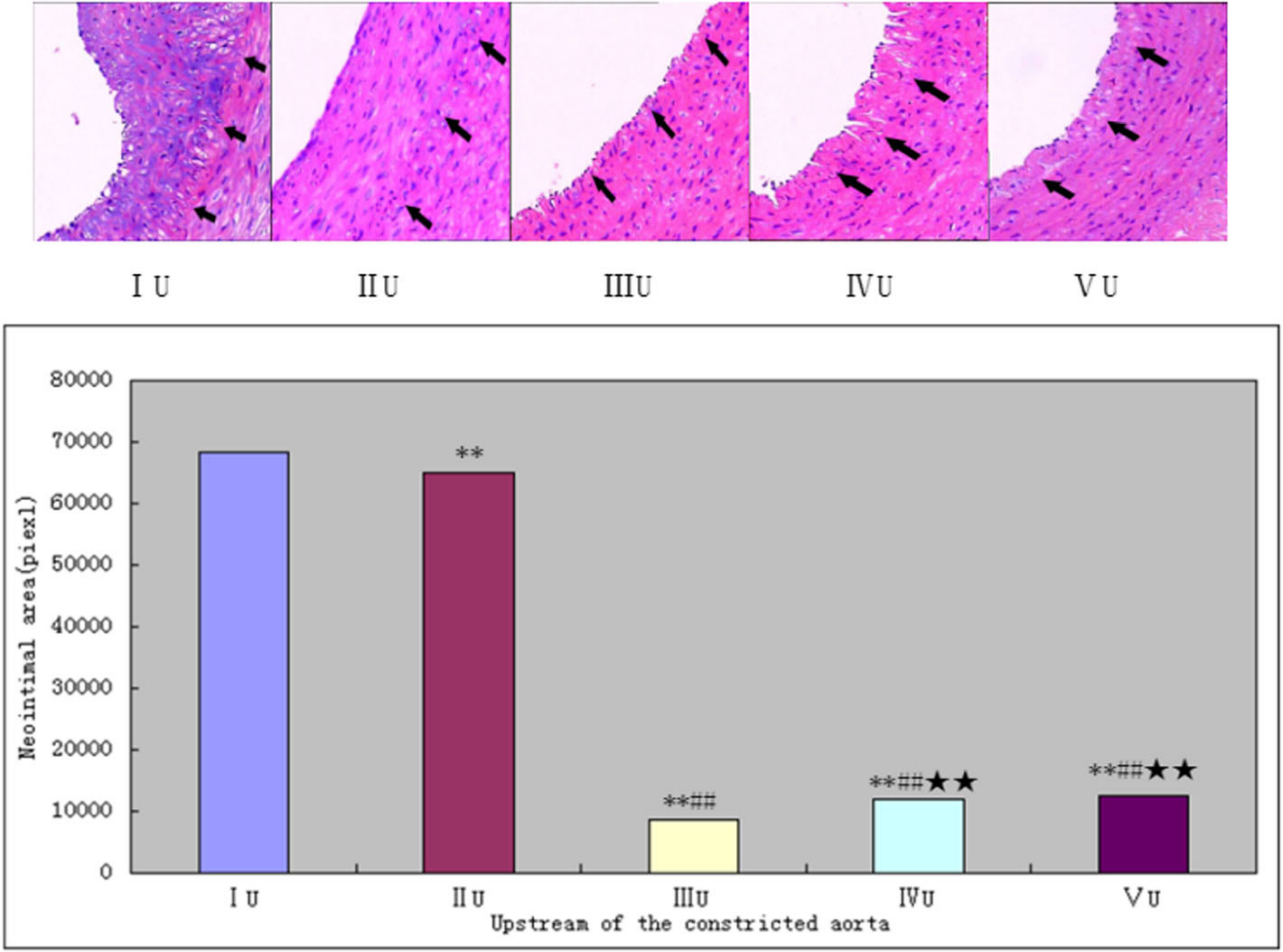
The upstream vascular neointimal area of the constricted aorta was evaluated by HE 4 weeks later (200×). Internal elastic lamina was shown by the arrows. **P < 0.05 significantly different from the groupIU; ##P < 0.05 significantly different from the groupIIU;★★P < 0.05 significantly different from the groupIIIU.U = upstream

The degree of neointimal hyperplasia was evaluated by morphologically and quantitatively after four weeks of the operation. In the upstream of the separated artery, similar to the results of the IVUS that balloon injury significantly increased the neointimal area in group IU (68,335 ± 3867, pixel, Fig. 5, ^**^ *P* < 0.05) and group IIU (64,851 ± 3995, pixel, Fig. 5, ^##^ *P* < 0.05) compared with other no balloon injury groups. Hemodynamic changes caused by artery constriction didn’t aggravate the neointimal hyperplasia which was secondary to the balloon injury, but attenuate the neointimal reactive hyperplasia in group IIU compared with group IU (^**^ *P* < 0.05). However, compared with the group IIIU (8418 ± 737, pixel), constriction significantly enhanced neointimal hyperplasia at the normal artery in group IVU (11,729 ± 1205, pixel, ^★★^ *p* < 0.05) and in group VU (12,338 ± 1240, pixel, ^★★^ *p* < 0.05), while this change wasn’t reflected by IVUS measurement.

**Fig. 6:**
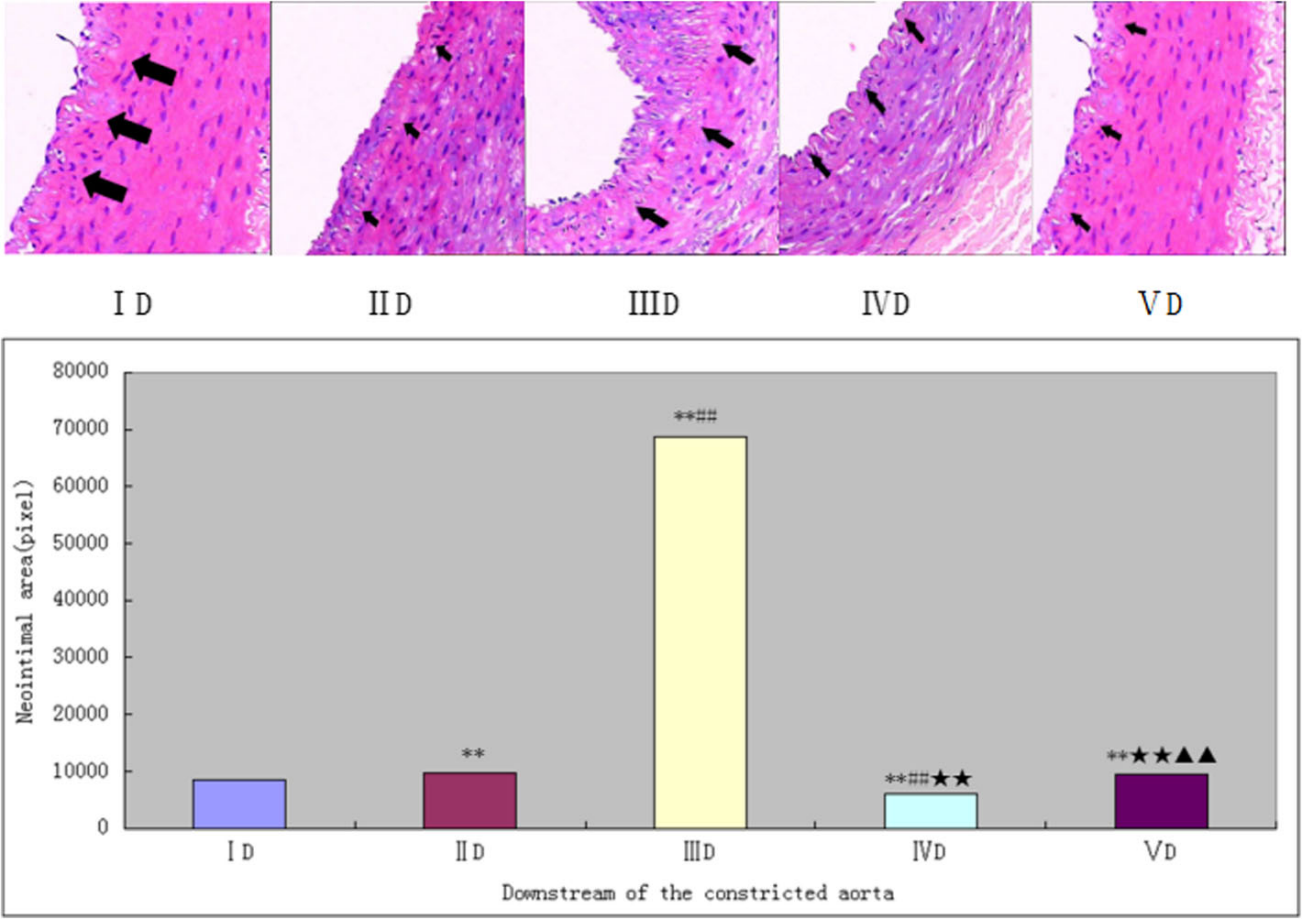
The downstream vascular neointima area of the separated artery was evaluated by HE after 4 weeks (200×). Internal elastic lamina was shown by the arrows. **P < 0.05 significantly different from the groupID; ##*P* < 0.05 significantly different from the groupIID;★★*P* < 0.05 significantly different from the groupIIID;▲▲*P* < 0.05 significantly different from the groupIVD.D = downstream

In the downstream of the separated artery, simple constriction can cause the downstream artery has a significantly neointimal hyperplasia in group IID (9628 ± 262, pixel,) and VD (9532 ± 324, pixel) compared with group ID (8530 ± 316, pixel; ^**^ *P* < 0.05), but the lumen diameter is increase according to the IVUS. Balloon injury made the intimal hyperplasia significantly in group IIID (68,750 ± 1617, pixel, Fig. 6, ^★★^ *p* < 0.05) compared with other all groups, however, constriction didn’t aggravate the neointimal hyperplasia but caused the intimal almost completely disappeared in group IVD (5950 ± 632, pixel, Fig. 6, ^▲▲^ *P* < 0.05), and the lumen diameter was excessive expansion compared with all other groups.

### The upstream SS of stenosis accelerated re-endothelialization after balloon injury, but in contrary to the effects of downstream SS

**Fig. 7:**
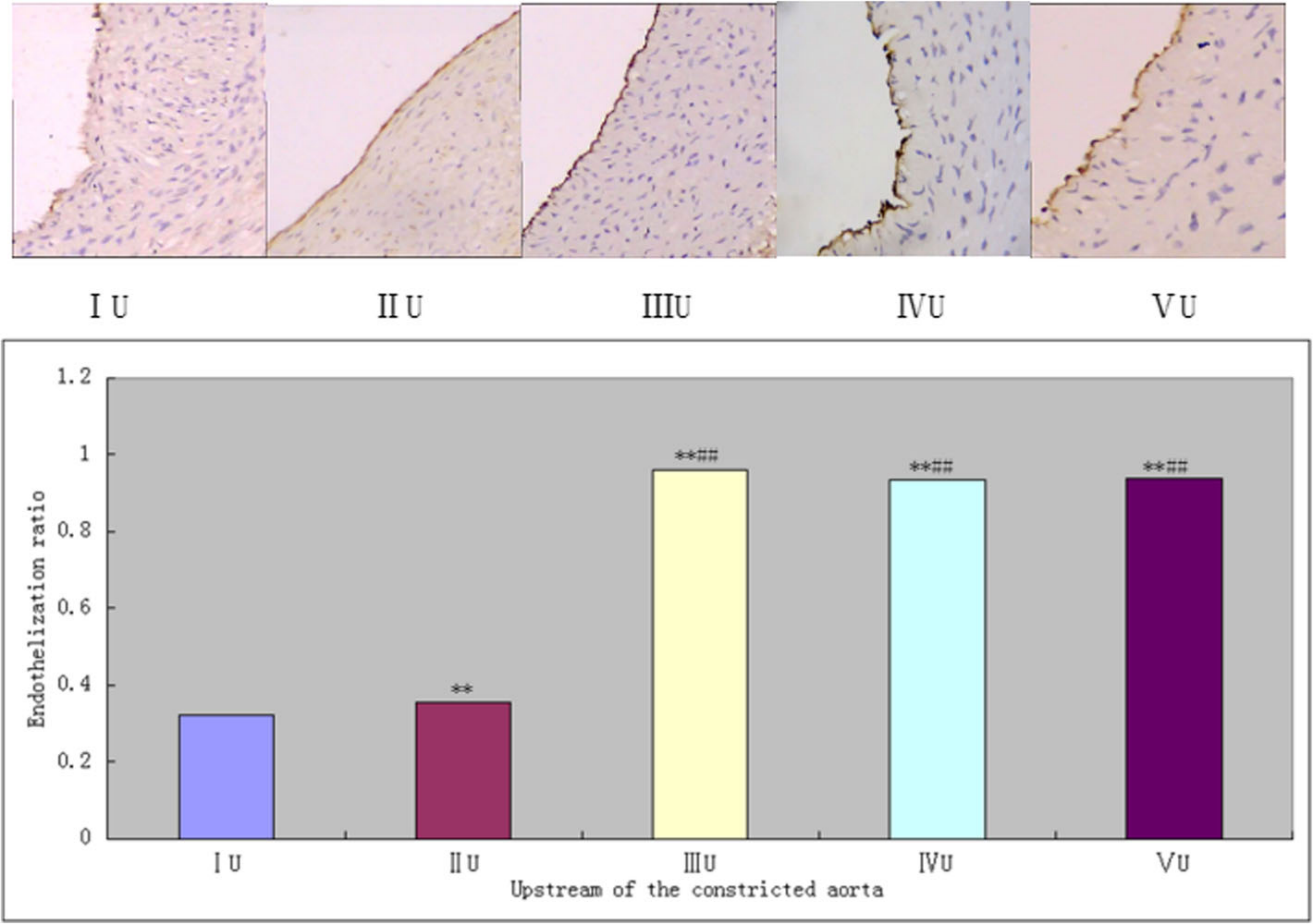
Effects of vascular stenosis and balloon injury on re-endothelialization in the upstream of the constricted abdominal aorta in each group (200×). Immunohistochemical staining for CD31 was performed and the ratio of re-endothelialization was evaluated. Typical observations of endothelial cells stained by anti-CD31 antibodies in the upper panel and the re-endothelialization ratio quantified in the lower panel. **P < 0.05 significantly different from the groupIU; **P < 0.05 significantly different from the groupIIU.U = upstream

**Fig. 8:**
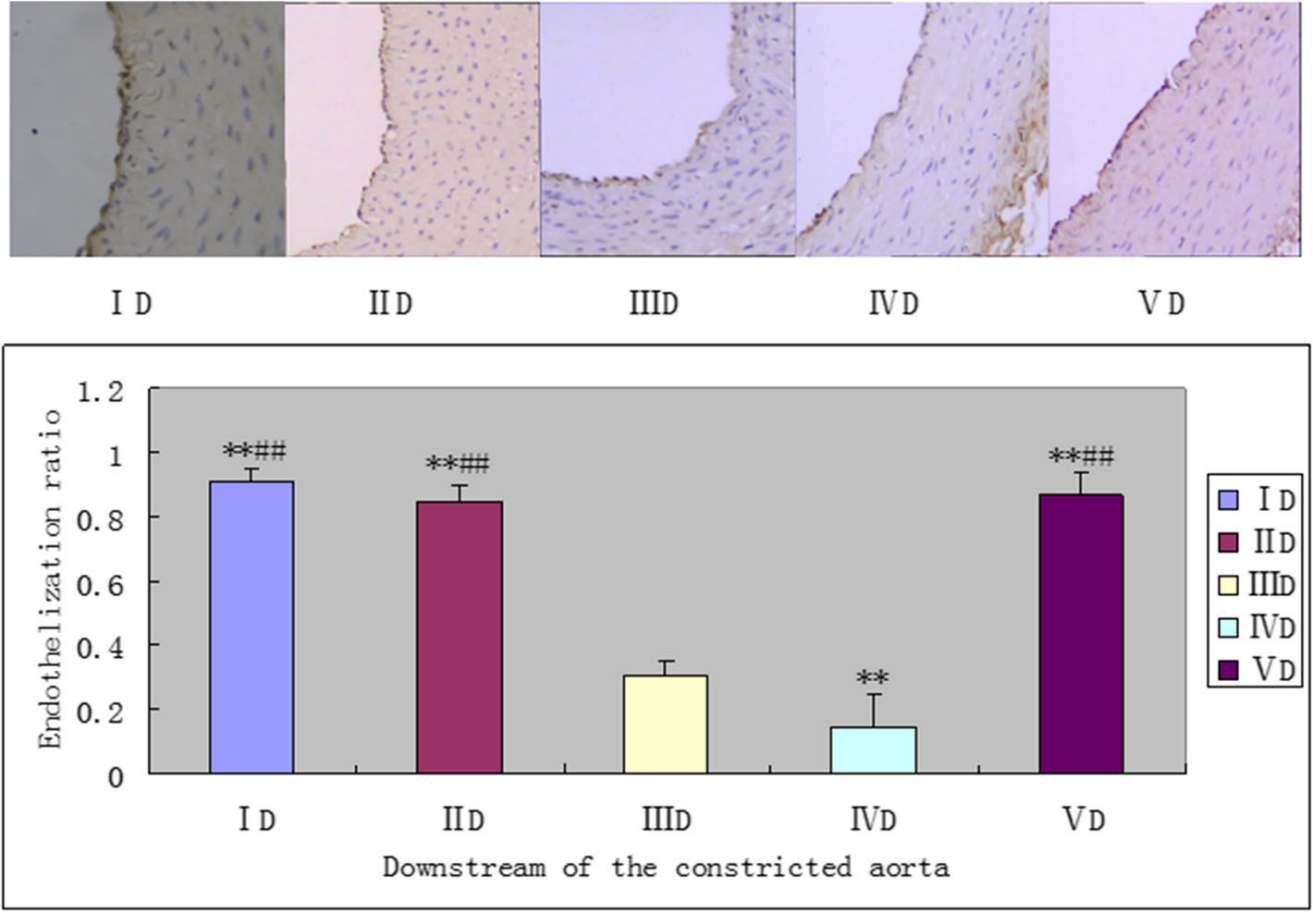
Effects of vascular stenosis and balloon injury on re-endothelialization in the downstream of the constricted abdominal aorta in each group (200×). Immunohistochemical staining for CD31 was performed and the ratio of re-endothelialization was evaluated. Typical observations of endothelial cells stained by anti-CD31 antibodies in the upper panel and the re-endothelialization ratio quantified in the lower panel. **P < 0.05 significantly different from the groupIIID; **P < 0.05 significantly different from the groupIVD.D = downstream

To investigate the effects of low ESS on re-endothelialization of the injured artery, the endothelial cell marker (CD31) was assessed in the immunohistochemical staining with the anti-CD31 antibody. Re-endothelialization was calculated as the ratio of the surface covered by CD31- positive cells to the total luminal surface. As shown in Fig. 7 (IIIU) and Fig. 8 (ID), CD31 positive immunostaining was observed along the luminal surface of the vessel wall. The upstream SS accelerated re-endothelialization of the balloon injury artery was observed in the group IIU compared with the group IU (Fig. 7, *P* < 0.05), but showed incomplete and sparse endothelium in both groups. However, the changed SS caused by artery constriction can undermine the integrity of the endothelial in the no balloon injury groups (VIU and VU) compared with the group IIIU, but no statistical significance was observed. In contrast to the upstream SS, the downstream SS inhibited the repair of injured endothelial cell in group VID compared with group IIID (Fig. 8, *P* < 0.05), and re-endothelial ratio in this group was significantly lowered than the group ID (Fig. 8, *P* < 0.05).

## Discussion

SS is a biomechanical force generated by fluid flow on the surface of the endothelium and involved in all stages of vascular physiology or diseases [6]. However, the effect of disturbed SS, induced by vascular constriction after the balloon injury, on the neointimal hyperplasia has not been fully understood in vivo. Cheng et al*.* [7] has established a perivascular SS model in mouse carotid arteries which exposed vascular upstream regions under influence of low and unidirectional SS, while downstream under low and oscillatory (ie, with vortices) SS, and they explore the plaque formation and composition with these models.

**Fig. 9:**
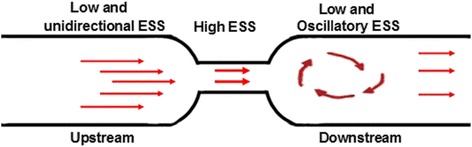
Schematics of the proposed mechanisms for disturbed blood flow in both upstream and downstream

Thus, on the basis of previous studies [6, 8], we established this animal model to explore the combination of changed SS and balloon injury results in neointima hyperplasia approximating that seen in clinical arterial restenosis (Fig. 9). The main findings of this study are as follows:(1) In the upstream of the stenosis, the proximal SS can cause the normal endothelial cell dysfunction and lead to the neointima hyperplasia but accelerated post-injury vascular re-endothelialization so as to attenuate neointima hyperplasia; (2) In the downstream of stenosis, the distal SS leads to the normal vessel outward remolding and this expansion tendency becomes more apparent if the integrity of endothelial cells was destroyed.

The primary sensors of mechanical forces in the vessel wall are endothelial cells. A considerable amount of literature on EC responses to SS has documented about EC morphology [9], function [7], and gene expression [1], and on the differentiation of immature cells, such as endothelial progenitor cells (EPCs) and embryonic stem (ES) cells [10]. As a primary enzyme that produces nitric oxide (NO), endothelial nitric oxide synthase (eNOS) mainly regulated blood vessel relaxation and its expression could be influenced by various mechanical forces, including SS [7, 11]. Re-endothelialization is beneficial to suppress neointimal hyperplasia with vascular endothelial growth factor (VEGF) coated stents after balloon injury [12]. In our study, we demonstrated that the upstream SS undermines the integrity of the endothelial cells, which further impact the function of endothelial cell and vascular homeostasis, thus lead to the neointimal hyperplasia in normal vessel. Moreover, the proximal SS inhibited the neointimal hyperplasia after balloon injury by means of accelerating re-endothelialization of the injured artery, but this effect was not maintained in the downstream SS.

Previous study showed that disturbed flow of a step-flow channel damaged ECs more severely than blood flow in static conditions [13]. In contrast, a high level of laminar SS inhibited proliferation of ECs [1] via reducing DNA synthesis [14]. Thus, the models of low SS leads to early neointimal hyperplasia probably via more complicated mechanisms, including enhanced SMC migration or proliferation, upregulation of multiple factors (VCAM-1, PDGFs, MMP-9), aberrant monocyte adhesion and uncontrolled macrophage infiltration [1]. Restriction of the common carotid artery (CCA) by ligating the distal CCA was performed in previous study to mimic low SS, and low SS in this model significantly increased the neointimal thickness after 4 weeks CCA ligation [15].This point was also demonstrated in our study that on the upstream and downstream of stenosis, neointimal hyperplasia was found at the low laminar SS district in the normal vessel. Accordingly, our animal model, by narrowing the vessel instead of reducing blood flow, can more accurately mimic hemodynamic changes caused by artery stenosis, such as atherosclerosis plaque in clinical scenario.

The structural integrity and functional completeness of the endothelial monolayer directly influenced vascular homeostasis. As a common physiological process in multiple vascular diseases, vascular remodeling responds to long-term effect of disturbed hemodynamic conditions, and further contributes to the pathophysiology progress of intima-media thickening (IMT), which accelerated progress of vascular diseases [16]. The effect of SS upon endothelium is a well-kown determinant of vascular remodeling. For instance, vessel diameter was abnormally increased by disturbed blood flow acceleration attributable to an arteriovenous shunt. Conversely, blood flow reduction results in the cascade reaction of signaling pathways which lead to atrophy of target vascular lumen [17]. In our study, the downstream low, oscillatory (ie, with vortices) SS leads to outward remolding with predominantly increased lumen and attenuation in the external elastic lamina, the reason may be the changing SS to achieve a homeostasis balance.

Although there has been evidence that low SS was involved in the pathophysiologic process of restenosis [18], but there are also reports that successful balloon angioplasty induced high SS which contributed to restenosis and target lesion revascularization, or during the follow-up period [4]. These seemingly contradictory conclusions illustrate the complexity of SS on the role of neointimal hyperplasia [19]. Chow et al. showed that pathological levels of SS (>3 N/m^2^) initiated platelet aggregation [20]. This view also can be used to explain our finding in the upstream of stenosis, in which low unidirectional SS inhibits the activation, deposition of platelet and weaken the neointimal hyperplasia ultimately.

## Conclusion

Appropriate low unidirectional SS is beneficial for the inhibition of intimal hyperplasia after balloon angioplasty, and it is a potential management target of in-stent stenosis.
